# Dentin degradonomics – The potential role of salivary MMP-8 in dentin caries

**DOI:** 10.4317/jced.56144

**Published:** 2020-02-01

**Authors:** Ajay Ashwini, Thayalan Dineshkumar, Annasamy Rameshkumar, Raman Swarnalakshmi, Ahmed Shahnaz, AE Nagarathinam, Krishnan Rajkumar

**Affiliations:** 1 Post Graduate Student, Department of Oral Pathology and Microbiology, SRM Dental College, SRM Institute of Science and Technology, Chennai, Tamil Nadu, India; 2 Associate Professor, Department of Oral Pathology and Microbiology, SRM Dental College, SRM Institute of Science and Technology, Chennai, Tamil Nadu, India; 3 Professor, Department of Oral Pathology and Microbiology, SRM Dental College, SRM Institute of Science and Technology, Chennai, Tamil Nadu, India; 4 unior Research Fellow, Department of Oral Pathology and Microbiology, SRM Dental College, SRM Institute of Science and Technology, Chennai, Tamil Nadu, India; 5 Professor and Head, Department of Oral Pathology and Microbiology, SRM Dental College, SRM Institute of Science and Technology, Chennai, Tamil Nadu, India

## Abstract

**Background:**

Dentin caries involves dissolution of minerals which eventually leads to degradation of organic matrix. This degradation which was thought to be by bacterial proteases is now considered to be orchestrated by endogenous collagenases such as Matrix Metalloproteinases (MMPs). This paper aims to estimate the salivary levels of MMP-8 in dental caries and also to asses the various risk factors that contribute to the formation of dental caries.

**Material and Methods:**

A random sample of 75 adults aged 18-35 were included and categorized based on the number of caries lesions (MCL). Standard clinical examinations were performed, and stimulated saliva was collected and analyzed for concentrations of MMP-8 using enzyme-linked immunosorbent assay. Caries risk factors were assessed using a chair-side kit. Correlation of MMP-8 in varying MCL using Spearman’s correlation was done. Multiple linear regression analysis was done to asses the relationship between various caries risk factors with MMP-8 and MCL as dependent variable.

**Results:**

The study results showed a statistically significant higher concentration of MMP-8 in carious group (MCL 1-2) and MCL ≥3 compared to non-carious group. On correlating, the levels of MMP-8 were seen to be higher in MCL ≥3 than in MCL = 0 and MCL 1-2. The mean MMP-8 of controls, MCL 1-2, and MCL ≥3 were 131.34ng/ml, 230.14ng/ml, and 391.91ng/ml respectively. Multiple linear regression analysis with MMP-8 as the dependent variable revealed caries, buffer capacity and *S. mutans* count as significant variables. Using MCL as the dependent variable the only significant variable was MMP-8 levels.

**Conclusions:**

The study suggests that subjects with caries have elevated MMP-8 levels compared with subjects with no carious lesions. There is also a positive correlation between the number of carious lesions and MMP-8 levels suggesting that MMP-8 plays an important role in the degradation of dentin and causes progression of caries.

** Key words:**Caries, Dentin, Enzyme-linked Immunosorbent Assay, MMP-8.

## Introduction

Dental caries (DC) is one of the most common chronic oral disease that is recognized as the primary cause of pain and tooth loss. DC is a progressive and irreversible disorder with a multifactorial etiology that requires a cariogenic bacteria to act on a suiTable substrate (1). It is a world wide-health problem affecting both primary and permanent dentition with increasing incidence, prevalence and high costs of treatment consuming about 5–15% of health care budgets (2).

It is estimated that an average of 2.43 billion people are affected by caries and is the principal oral health concern which hinders the achievement and maintenance of oral health across all age groups (1). Inspite of increasing knowledge about the etiology and pathogenesis of the disease, it is still responsible for high morbidity rates and is associated with decreased quality of life. Recent statistics from the 2015 “Global Burden of Disease Study” showed that dental caries is the the most prevalent condition giving it a distinction of ranking first for decay of permanent teeth and 12th for deciduous teeth (2,3).

Dental caries is a post-eruptive irreversible microbial disorder characterized by progressive demineralization of inorganic substance by the action of acids produced from fermentation of dietary carbohydrates. This hard tissue loss that occurs in dentin is due to dissolution of dentinal minerals that eventually leads to degradation of the collagenous organic matrix. However, the precise mechanism behind collagen matrix degradation is obscure and over the years microbial proteolytic enzymes were believed to be responsible for degradation of dentin organic matrix. However, this concept has been challenged as there is substantial evidence to prove that the oral microbiome does not possess the proteolytic competency to bring about degradation of intact collagen (4).

As early as 1983, Dayan *et al.* demonstrated endogenous collagenolytic activity orchestrated by proteases in carious dentin, however this concept was ignored owing to limited knowledge of proteases (5). With increasing insight of these proteases over the years, it is now considered that degradation of dentin occurs as a result of endogenous proteases that are present in the dentin and has paved way for a new concept in the field of caries research termed as “Dentin Degradonomics”. Degradonomics is a process of genomic and proteomic approach to identify, proteases and its substrates in both physiological and pathological conditions (6,7).

Recent data have suggested the role of endogenous Matrix Metalloproteinases (MMPs) in the process of dentin destruction following demineralization by the acid attack initiated by bacteria. MMPs are a family of Calcium and Zinc dependent enzymes, which have become biologically important because of their ability to practically degrade all extracellular matrix components. It is proposed that these MMPs are a product of odontoblasts and are involved in the formation of dentin. Once collagen matrix mineralization occurs, these MMPs remain entrapped in the calcified matrix as inactive proforms and are re-exposed and potentially activated by an acidic ambiance created by bacterial acids and ultimately cleave the matrix components resulting in degradation of dentin. Hence, pH changes characteristic of carious lesions act as potent MMP activators (8). Of the various MMPs studied, MMP-8 is considered to be the major collagenolytic enzyme in dentin and is found predominantly in the outer portions of the dentin compared to the deeper portions (9). Combined with its relative high presence in outer portions of carious dentin and its efficient catalytic capability to digest type I collagen, MMP-8 is considered to play a pivotal role in dentin matrix degradation.

Over the last few years, MMPs have also gained importance and have made giant strides in the field of restorative dentistry. Ever since the foundation laid by Buonocore in 1955 in the field of dental adhesives, the hybrid layer always remained the weakest link in maintaining adequate bond strength (10). Despite all the improvements in adhesive systems it always remained a potential challenge, until recently it was proposed that MMPs are key mediators for maintaining adequate bond strength. It was postulated that when the dentinal endogenous MMPs are exposed and reactivated by the etching process, it leads to degradation of the type I collagen fibrils and in turn influences the bond strength (11).

Caries is regarded as a multifactorial disease wherein it is influenced by a variety of environmental, behavioural and biological factors, which, if present, frankly will increase the chance of disease occurrence. These factors are most commonly referred to as “caries risk factors” and these factors are a part of the pivotal chain, or they can also expose the host to the causal chain. Thus caries risk assessment is the first phase in management of dental caries and the level of risk should be assessed and used to determine the need for therapeutic intervention and is an important part of treatment planning.

Considering the above facts and the lack of clinical data regarding the role of proteolytic enzymes in dentinal caries progression, this study aims at evaluating one of the promising proteolytic enzymes, MMP-8 to better understand the pathogenesis behind dentin degradation and also to assess the relationship of various caries risk factors (salivary secretion rate, pH of saliva, Buffering capacity and *Streptococcus mutans* count) for better therapeutic intervention and appropriate treatment planning.

## Material and Methods

A prospective case control study was conducted in SRM Dental College, Ramapuram, Chennai. During the period of 2017 January to 2018 February, patients attending the outpatient department were recruited after obtaining ethical clearance (SRMDC/IRB/2016/MDS/No.601) and verbal and written consent. Saliva samples are collected from a total of 75 subjects with age range of 18 to 35 years, out of which the control group (No caries - Group I) comprised of 25 subjects and the study group (Caries Group - Group II) had a total of 50 subjects, which were further categorized based on the number of caries lesions referred to as Manifest Caries Lesions (MCL) into Group IIa (MCL 1-2) consisting of 25 subjects and Group IIb ((MCL ≥ 3)) consisting of 25 subjects.

-Study Groups

Group I- Control Group (No caries) - 25

Group II- Caries Group (caries involving dentin) - 50

Group IIa (MCL 1-2): 25 (Caries not involving more than 2 teeth)

Group IIb (MCL ≥ 3): 25 (Caries involving more than 3 teeth)

-Clinical Examination

A thorough standard clinical examination was performed, as well as radiographic examination including digital bitewing and panoramic radiography and subjects were allotted in the appropriate group. Complete clinical examination and saliva sampling was done for all patients and were analysed for the following:

• Decayed Missing Filled Surface (DMFS) and Oral Hygiene Index-Simplified (OHI-S) index of all subjects

• Saliva flow rate, pH of saliva and Buffering capacity of saliva using Saliva Check Buffer Kit - GC©

• *Streptococcus mutans* count by culture method using Mitis Salivarius Bacitracin Agar (MSB)

• Concentration MMP-8 in saliva of all the groups using ELISA

-Saliva sampling and chair-side analysis 

Stimulated saliva was collected after chewing on the paraffin wax for 5 minutes. After 1 minute patient expectorated all the saliva into a graduated tube and the secretion rate was determined and expressed as milliliter/minute. Salivary buffer capacity and pH of saliva were determined with Saliva Check Buffer Kit-GC©. Chair side kit was obtained from GC Asia and handled according to the manufacturers instructions. Each individual was again asked to expectorate the saliva into the container at the end of 5 minutes. Samples were centrifuged at 2500 rpm for 15 min at 40c and the supernatant separated into 1 ml aliquots and stored at –800c for further analysis.

-*Streptococcus mutans* count

Mitis Salivarius Bacitracin Agar (MSB) was used for the selective isolation of streptococcus mutants (SM). Salivary samples were diluted in 0.05 M phosphate buffer (pH 7) to dilutions of 10-2 and 10-3 and agitated for 30 seconds and were inoculated onto the medium. Cultures were grown under suiTable environment of 5% carbon dioxide and 95% nitrogen and was incubated for 18 hours. Ten-fold dilutions were made in 9 ml phosphate buffer at a pH of 7.2. Duplicate samples were plated as 1 in 10 (1/10), 1 in 100 (1/100) and 1 in 1000 (1/1000) dilutions and spread on the surface of the medium using surface plating method. The plates were allowed to cool at room temperature for 24 hours and cultural characteristics were observed after incubation at 35-370C for 18-48 hours. SM colonies were recognized on the MSB agar plate by their morphology, appearing creamy white in colour, round/spherical/raised, ranged from pinpoint to pin head size with a rough surface. A Digital Colony Counter was used to examine the plates and count the colonies formed and expressed as x105 cfu/ml.

-Estimation of MMP-8 concentration

Quantification of salivary MMP-8 was done by commercially available ELISA kit (Bioassay Technology) which is based on Biotin antibody sandwich technology. The assay was carried out according to the manufacturer’s instructions. Briefly, to the precoated MMP-8 antibody microplate, standards and samples were added, incubated and washed with buffer. To the washed microtiter plate detection antibody bound with HRP conjugate was added. The unbound antibody was washed and a chromogen substrate was added to the wells resulting in the progressive development of a blue coloured complex with the conjugate. The reaction was then stopped by the addition of stop solution turning the resultant final product yellow. The intensity of colour developed is proportional to the MMP-8 present in the sample which was measured in a microplate reader at a wavelength of 450nm. The optical density obtained was then used for calculation of MMP-8 present in each sample.

-Statistical analysis

The data collected was entered into Microsoft excel spreadsheet. Statistical analysis of the data obtained was done using SPSS software (version 22). Descriptive statistics such as mean and standard deviations (SD) for all parameters were calculated for individual groups. Student’s t-Test was used to statistically analyze the level of significance between each group and for analysis within groups one-way ANOVA was applied. Spearman’s correlation analysis was applied to analyze the correlation between salivary MMP-8 levels and dental caries. Linear regression analysis was done to assess the perusal of relationships between the variables. A *p-value* of less than 0.05 was considered to be statistically significant.

## Results

The mean values showed a statistically significant difference (*p*<0.05) in the salivary MMP-8 in subjects with caries compared with subjects without caries and the mean salivary levels of MMP-8 in controls was 131.34ng/ml, while the concentration of MMP-8 in MCL1-2 and MCL≥3 were 230.14 and 391.91ng/ml respectively ( Table 1). Also there was a strong positive correlation between MMP-8 and MCL ( Table 2). Caries risk assessment parameters were done and the differences between the MCL groups were statistically significant for all the predictive variables ( Table 1, Table 3).

**Table 1:**
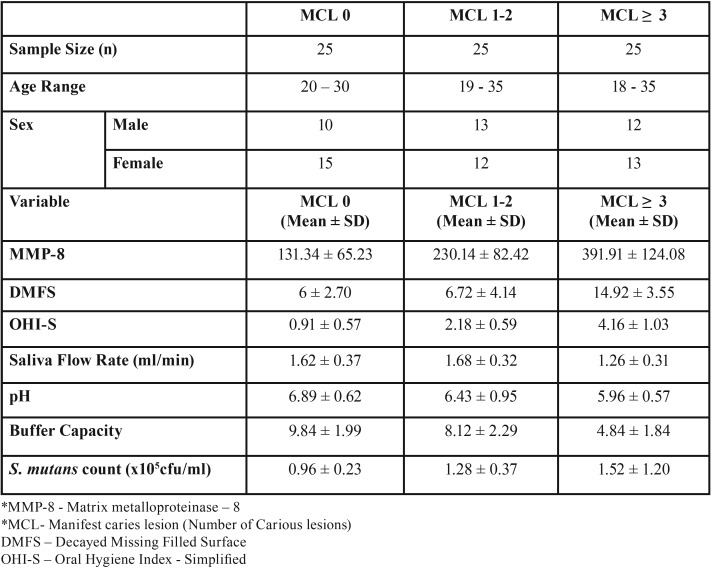
Descriptive statistics for salivary MMP-8* and all predictable variables by MCL* in caries and control group.

**Table 2:**
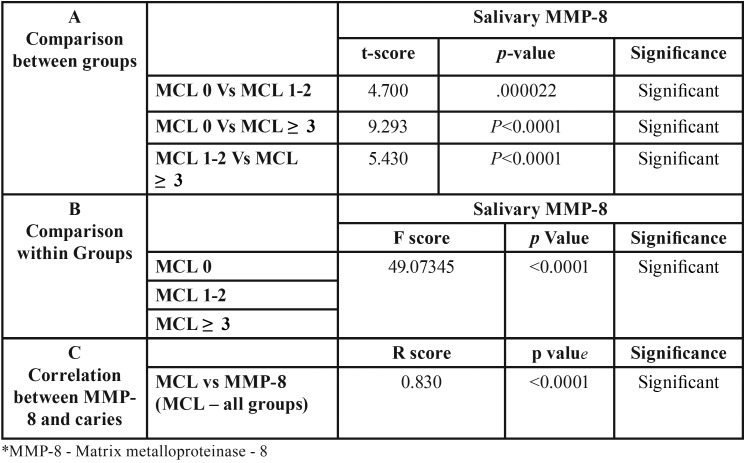
Comparison and Correlation MMP-8 between groups.

**Table 3:**
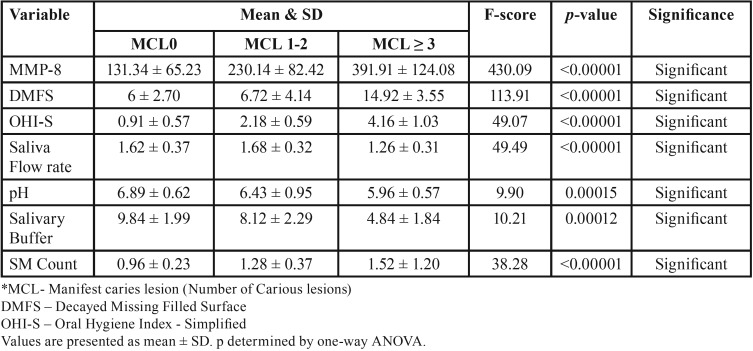
Statistical Analysis of predictable variables based on MCL.

To determine the impact of the various variables on the MCL score a multiple linear regression analysis was performed ( Table 4) with MMP-8 (R2 = 0.785) as the dependent variable, the statistically significant predictor variables were MCL, Salivary buffer capacity and Salivary mutans count. Using MCL (R2 = 0.845) as the dependent variable the predictor variables MMP-8, DMF-S and OHI-S were statistically significant.

**Table 4:**
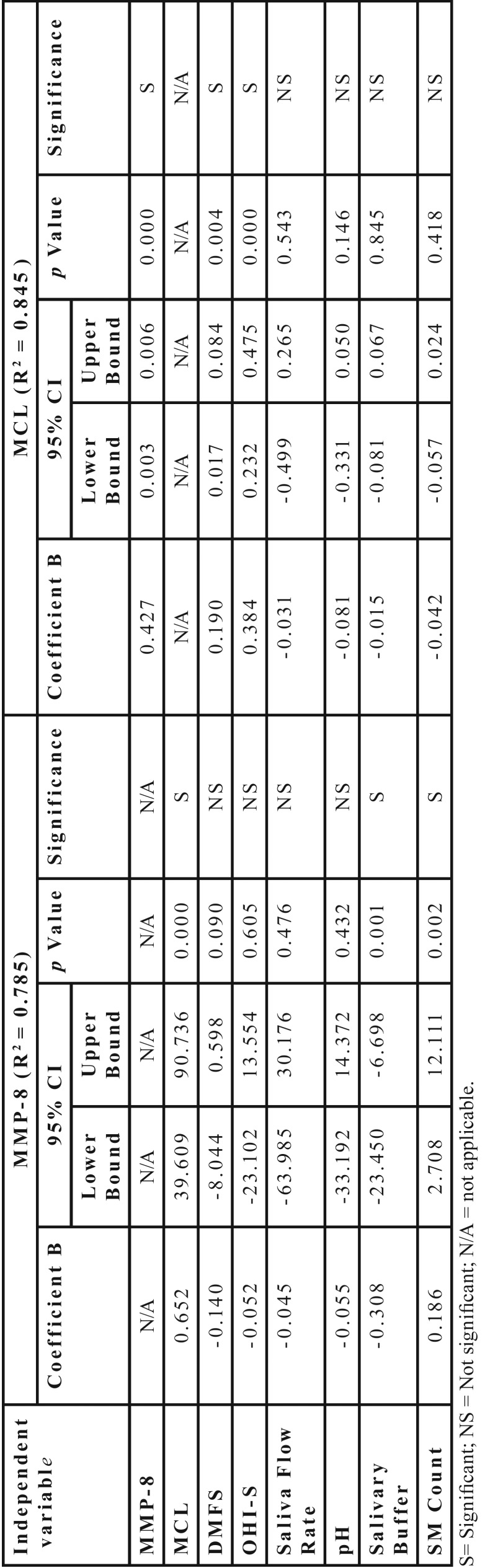
Multiple Linear regression analysis with MMP-8 and MCL as dependent variables.

## Discussion

Being one of the most common chronic disease, Dental Caries culminates as an irreversible localized loss of mineralized structures with subsequent dissolution of the organic content of the tooth (12). During this initial demineralization phase, the hydroxyapatite crystals are dissolved by the acids generated by bacteria and the acid diffuses into the calcified tissues when there is a local drop in the pH, leading to further dissolution of crystals (4). In dentin, this degradation of collagen matrix was long speculated to be the role-played by bacterial proteases (13).

However, it is now shown that cariogenic bacteria do not possess the repertoire to degrade dentin matrix after initial demineralization. Since there was a lack of evidence for a bacterial role in the degradation of matrix collagen in carious dentin, researchers believe it to be orchestrated mainly by a family of host-derived endogenous proteases called as matrix metalloproteinases (MMPs) (14).

The MMPs present in dentin are thought to play a role in dentinogenesis and are produced by the odontoblasts during secretion of dentin matrix. Once collagen matrix mineralization occurs, the MMPs remain entrapped within the calcified matrix as inactive forms, only later to be unmasked and switched on during a caries attack in the dentin. This triggering of endogenous MMPs are promoted by the acidic environment created by the bacteria. Although the pH-activated MMPs are sTable in an acidic environment, they function best at neutral pH, which is brought about by the buffering systems of the saliva, thus enabling the energized MMPs to break down organic matrix components (15).

An array of MMPs have been shown to be actively operating in the course of carious process including MMP-2,8,9,20. However, it is shown that MMP-8 operates as a true collagenase and is most efficient in hydrolyzing type I collagen (16). Even though, dentinal fluid serves as a primary source of MMPs in caries, it is also possible that saliva could be an additional contributor to these proteases aiding in the matrix degradation process (15). Several MMPs are reported in saliva apparently derived from salivary glands and gingival crevicular fluid out of which MMP-8 and MMP-9 are hypothesized as the abundant and predominant proteases in dentin caries especially in the outer layers of caries compared with the deep caries layer (16,17).

Collectively, the above findings suggest saliva as an important contributor of these proteolytic enzymes and it is logical to hypothesize that salivary MMPs come in proximity with dentin during a caries attack and contributes to the carious process in dentin by collagen matrix degradation.

Although current literature suggests that salivary MMPs play an important role in the degradation process, it is not without their limitations. One of the important drawback is that most of these studies are carried out *in vitro* and extrapolating these results would not be a true indicator of its role in a clinical setting, as dental caries is a disorder of multifactorial etiology. Hence, to throw more light on the role played by salivary MMPs, this study was conducted with a primary objective of estimating and correlating the salivary levels of MMP-8 and dental caries. Also, known caries risk factors such as salivary flow rate, pH, buffering ability of saliva and streptococcus mutans count were also investigated.

-MMP-8 estimation and correlation 

The study results showed increased concentration of salivary MMP-8 in subjects with caries compared with subjects without caries irrespective of age and gender. The mean salivary levels of MMP-8 in controls was 131.34ng/ml, while the concentration of MMP-8 in MCL1-2 and MCL≥3 were 230.14 and 391.91ng/ml respectively.

The results also indicate that there was a steady and progressive increase (2 to 3 fold) in the salivary levels of MMP-8 from subjects with no caries to subjects with few or many caries lesions and it was found to be statistically significant. Subsequently, to find out if there was any correlation between caries and salivary MMP-8 levels, correlation analysis was done and a strong positive correlation was observed.

The results were in accordance with Hedenbjörk-Lager *et al.* who showed similar elevated levels of salivary MMP-8 level in subjects with caries in comparison with no caries subjects. Furthermore, they also demonstrated an elevated MMP-8/TIMP-1 ratio, suggesting an imbalance between the enzyme and its inhibitor (6).

Gursoy *et al*. showed a similar elevated MMP8/TIMP-1 ratio in periodontal disease and suggested that the amount of tissue destruction is an after effect of the imbalance between the enzyme and its inhibitor, and this enzyme-inhibitor ratio can be utilized as a measure of proteolytic activity (18).

Studies to evaluate the *in vivo* activity of MMP in demineralized dentin are lacking and most are confined to measure MMP activity after treating dentin with self-etching adhesives which causes demineralization of dentin. This scenario may be comparable with the one occurring after demineralization during a carious attack.

Mazzoni *et al.* demonstrated a significant increase in MMP activity in partially demineralized dentin with phosphoric acid and their findings supported the role of endogenous MMPs in the degradation of hybrid layers (8).

Nishitani *et al.* indicated a 10-fold hike in gelatinolytic activity of mineralized dentin powder after application of self-etching adhesivs and suggested that a mild attack may activate dormant MMP and exacerbate the activity of MMP to maximum level thereby leading to degradation of dentin (19).

Shimada *et al.* using a novel gold colloidal labelling method investigated the localization of different MMPs-2,8,9&20 in normal and carious dentin. It was suggested that MMP-2 was scattered both in carious and normal dentin and its level did not show any difference among the normal dentin, outer and inner caries. However, the labelling indexes of both MMP-8 and MMP-9 showed a significant intensity at the outer caries region compared with that of inner caries and normal dentin (9).

These findings supported by our study results suggest the notion that saliva derived MMPs particularly MMP-8 may be more influential and vital than those from dentin for degradation of outer caries organic matrix.

-Assessment of caries risk factors 

The paradigm shift in the current understanding of the dynamic, multifactorial nature of dental caries and the resultant change in caries preventive and treatment strategies necessitates that caries risk assessment should be an integral part of any caries management protocol and hence becomes a vital element in the comprehensive management of dental caries.

Currently, caries risk assessment models comprise of “the risk model and the prediction model”. The risk model assess the causative factors referred to as the risk factors, but cannot predict the outcome whereas the prediction model estimates the risk of caries progression in the future.

Considering the importance of risk factor assessment, various risk factors were assessed including past caries experience, oral hygiene index (simplified), streptococcus mutans count, salivary flow rate, pH and buffering capacity of saliva.

Noticeable differences were found for all the individual risk factor variables between the groups of varying MCL and these differences were statistically significant. Although, significant when assessed individually, a regression model analysis was performed to assess the influence of the various variables.

With MCL as dependent variable and all other parameters as predictor variables, the only significant predictor variables were MMP-8 (*p* = .001) and OHI-S (*p* = .004). Similarly, with MMP-8 as dependent variable, the predictor variables MCL (*p* = .000), salivary buffer (*p* = .001) and streptococcus mutans count (*p* = .002) were statistically significant.

Collectively, the observed findings strongly suggest that salivary MMP-8 levels are elevated in subjects with caries in comparison with no caries. Also, estimation of such proteases can in future be a part of the caries risk assessment models and provide a guide to estimate the progression of caries.

However, this study is not without its own limitations. Although, elevated MMP-8 levels were present in the caries disease process, it is impossible to establish a cause-effect relationship. Despite our sincere efforts to strictly adhere to the inclusion and exclusion criteria, there could have been some form of gingival inflammation associated with proximal carious lesions. Hence, the estimated salivary MMP-8 could have originated from gingival crevicular fluid or from the carious lesion itself.

## Conclusions

The study results strongly suggest that subjects with caries have significantly elevated MMP-8 levels in comparison with subjects without caries. Also, caries correlated strongly with salivary levels of MMP-8 suggesting the potential role of MMPs in the progression of dental caries. Although, salivary MMP-8 levels were elevated, a true cause-effect relationship is difficult to establish as the MMPs itself could have originated from gingival crevicular fluid or from the carious lesions themselves. Therefore, further research needs to be carried out to establish a true cause-effect relationship between salivary MMP-8 and caries.

If the causal role of MMPs in dentin degradation is established, it could have huge implications in the field of dentistry including ; In Preventive Dentistry, MMP inhibitors could provide a therapeutic role in controlling or limiting the progression of caries in dentin. In Restorative Dentistry, traditional dentin adhesives lose their bond strength regardless of the bonding system used due to degradation of the dentin matrix with subsequent loss of mechanical properties of dentin leading to bonding failure. Hence, preservation of dentin collagen matrix becomes a prime issue in improving the dentin bonding durability. Hence, better understanding of the degradation of dentin matrix holds the future for designing an ideal dentin adhesive.
